# Structural Dynamics of the Precatalytic State of Human Cytochrome c upon T28C, G34C, and A50C Mutations: A Molecular Dynamics Simulation Perspective

**DOI:** 10.1021/acsomega.3c00220

**Published:** 2023-04-17

**Authors:** Bodee Nutho, Sasiprapa Samsri, Soraya Pornsuwan

**Affiliations:** † Department of Pharmacology, Faculty of Science, Mahidol University, Bangkok 10400, Thailand; ‡ Department of Chemistry and Center of Excellence for Innovation in Chemistry, Faculty of Science, Mahidol University, Bangkok 10400, Thailand

## Abstract

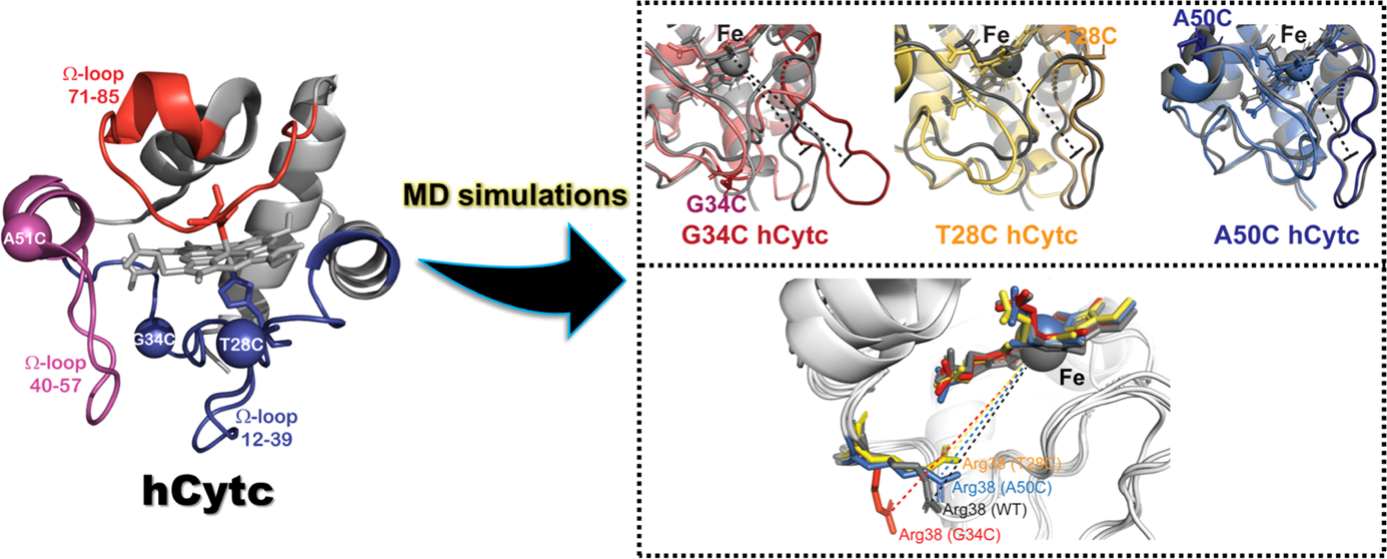

The native structure of cytochrome *c* (cytc) contains hexacoordinate heme iron with His18 and Met80 residues ligated at the axial sites. Mutations of cytc at Ω-loops have been investigated in modulating the peroxidase activity and, hence, related to the initiation of the apoptotic pathway. Our previous experimental data reported on the peroxidase activity of the cysteine-directed mutants at different parts of the Ω-loop of human cytc (hCytc), that is, T28C, G34C, and A50C. In this work, we performed 1 μs molecular dynamics (MD) simulations to elucidate the detailed structural and dynamic changes upon these mutations, particularly at the proximal Ω-loop. The structures of hCytc were modeled in the hexacoordinated form, which was referred to as the “precatalytic state”. The results showed that the structural features of the G34C mutant were more distinctive than those of other mutants. G34C mutation caused local destabilization and flexibility at the proximal Ω-loop (residues 12–28) and an extended distance between this Ω-loop region and heme iron. Besides, analysis of the orientation of the Arg38 side chain of the G34C mutant revealed the Arg38 conformer facing away from the heme iron. The obtained MD results also suggested structural diversity of the precatalytic states for the three hCytc mutants, specifically the effect of G34C mutation on the flexibility of the proximal Ω-loops. Therefore, our MD simulations combined with previous experimental data provide detailed insights into the structural basis of hCytc that could contribute to its pro-apoptotic function.

## 1 Introduction

Cytochrome *c* (cytc) is a well-known globular hemeprotein that performs multiple biological functions and has been categorized as a “moonlighting protein” due to its diverse binding targets.^1,2^ Specifically, the critical roles of cytc have been recognized as an electron shuttle in the electron transport chain and as a peroxidase to initiate the apoptotic pathway.^3,4^ In vivo, the pro-apoptotic function of cytc involves the interaction with cardiolipin (CL), a phospholipid located in the inner mitochondrial membrane.^5^ Upon binding to CL, the peroxidase activity of cytc is activated, leading to the oxidation of CL, causing the release of cytc into the cytosol where the caspase-dependent processes are triggered. The enhancement of peroxidase activity of cytc is, therefore, a crucial step in the pro-apoptotic pathway.

**Figure 1:**
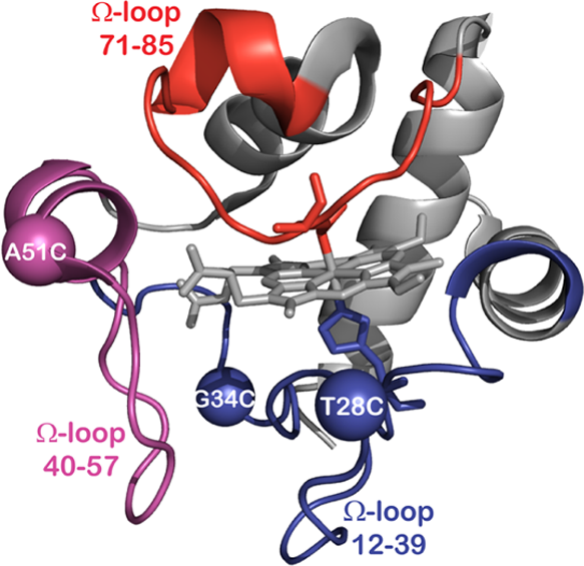
Crystal structure of the ferric WT hCytc (PDB ID: 3ZCF(25)) showing the mutated positions studied in this work, A50C (pink sphere), G34C, and T28C (dark blue spheres), and the three Ω-loop regions: proximal (residues 12–39 (dark blue)), central (residues 40–57 (pink)), and distal (residues 71–85 (red)) Ω-loops.

Structural characteristics of cytc are composed of ∼100–104 amino acids containing the heme moiety, which is hexacoordinated with four nitrogen atoms from the porphyrin ring as equatorial positions, and Met80 and His18 served as the axial coordinates. The protein fold consists of five α-helices connected by three Ω-loop regions defined as distal (residues 71–85), central (residues 40–57), and proximal (residues 12–39) Ω-loops^6^ (Figure 1). The native state of the cytc structure retains a low level of peroxidase activity due to the dominant hexacoordinate form of heme iron. The induction of peroxidase activity of cytc upon binding to CL has been established to be associated with structural rearrangements of the folding units, providing the disruption of the Fe-Met80 coordinate.^7^ This enables the pentacoordinate form that facilitates the access of substrates to the heme iron. Additionally, other structural modifications of cytc, such as post-translational modification^8^ and mutations, culminate in alternative conformations that support the increment of peroxidase activity. These include the naturally occurring mutations of human cytc (hCytc) at positions G41S, Y48H, and A51V.^9,10^ All are related to the pathophysiology of thrombocytopenia 4 (THC4), a disorder characterized by abnormally low counts of platelets. The findings have emphasized the important features of various ranges of conformational states of the protein that are closely relevant to biological functions. Identification of these alternative conformations to elucidate the structure–function relationships remains a major challenge.

Previous reports have focused on the observation of structures and dynamics of the distal Ω-loop by introducing mutations at the conserved residues in this Ω-loop.^11,12^ The study by Englander et al.^13,14^ and recent reports^15,16^ have shown that the flexibility at the distal 71–85 Ω-loop controls the ligand exchange behavior of cytc due to its direct contact with the Met80 residue. Further reports on the naturally occurring mutations on the 40–57 Ω-loop^17−19^ revealed that the conformational transitions at the distal Ω-loop, leading to a peroxidase active form, are controlled by kinetics of the central 40–57 Ω-loop. The stability of the central Ω-loop disturbs the dynamics in the distal 71–85 Ω-loop, as suggested by the increase of peroxidase activity of the naturally occurring mutants. Recently, our previous works aimed to explore the influence of the mutations at each Ω-loop on the peroxidase activity of hCytc. We have examined the cysteine-modified mutated hCytc at four random positions: T28C and G34C in the proximal Ω-loop, A50C in the central Ω-loop, and P76C in the distal Ω-loop.^20,21^ The advantage of preparing the cysteine side chain was the capability to examine the dynamics at the mutated site by spin labeling (SL) combined with electron spin resonance (ESR). Specifically, the ESR signals from the spin-labeled proteins allow us to directly determine the rotational correlation time, τ_R_, at the labeled sites.^22,23^ This conventional ESR approach is sensitive to the local flexibility due to molecular rotational motion and is suited for small compact proteins.^24^ Our previous experimental data^20,21^ showed that the flexibility at the site of mutations obtained from the ESR results was found in the following order: P76C > G34C > A50C > T28C hCtyc. This trend was consistent with the peroxidase activity of the proteins, where the activity of the WT was comparable to that of the T28C mutant (Table S1). Therefore, we further performed molecular dynamics (MD) simulations to predict the dynamic behavior of P76C (i.e., the most active peroxidase activity) and WT hCtyc in the hexacoordinate form referred to as the “precatalytic state”.^21^ The MD analysis on this state of the P76C mutant exhibited the weakening of the hydrogen bond between Tyr67 and Met80, which could be considered as an influence on the increase in peroxidase activity. Following our previous study, in this present work, we have extended the MD analyses on the mutants at proximal (T28C and G34C) and central (A50C) Ω-loops (Figure 1). With this regard, a long simulation time of 1 μs was carried out to provide detailed insights into the dynamics of the three additional mutants. The analysis of MD simulations was performed utilizing several structural parameters to elucidate the dynamic behaviors and relative peroxidase activities of each mutant in comparison with WT hCytc. Although the MD results on the trend of dynamics of the studied mutants do not agree well with those from the ESR and peroxidase activity, the MD data specifically revealed the precatalytic state of the G34C mutant that reflects distinctive dynamics at the proximal Ω-loop when compared to other mutants and WT hCytc (see details in Section 2). To our knowledge, the effect of mutations in the proximal Ω-loop on the structural changes is still underexplored. In particular, the different dynamics of the observed mutants could provide molecular insights into the multiple conformers of the pre-catalytic state of hCytc and the role of dynamics of individual Ω-loops contributing to apoptotic activity.

## 2 Results and Discussion

In this work, we propose that single mutations at various regions of Ω-loops would develop different conformations that relate to the modulation of peroxidase activity of hCytc. We thus continue to perform MD simulations on the T28C, G34C, and A50C mutants to obtain the structural dynamics of the precatalytic states of such mutants. Our MD results based on the calculations of structural parameters, including (i) root-mean-square displacement (rmsd), (ii) radius of gyration (Rg), (iii) solvent-accessible surface area (SASA), and (iv) root-mean-square fluctuation (rmsf), are described for each mutated system in comparison with those of WT hCytc. Besides, further analyses on distances between critical residues reflecting locally conformational flexibility and implication to peroxidase activity of each mutant are discussed in the following sections.

### 2.1 Structural Stability and Flexibility of the T28C, G34C, and A50C Mutants and WT hCytc

**Figure 2:**
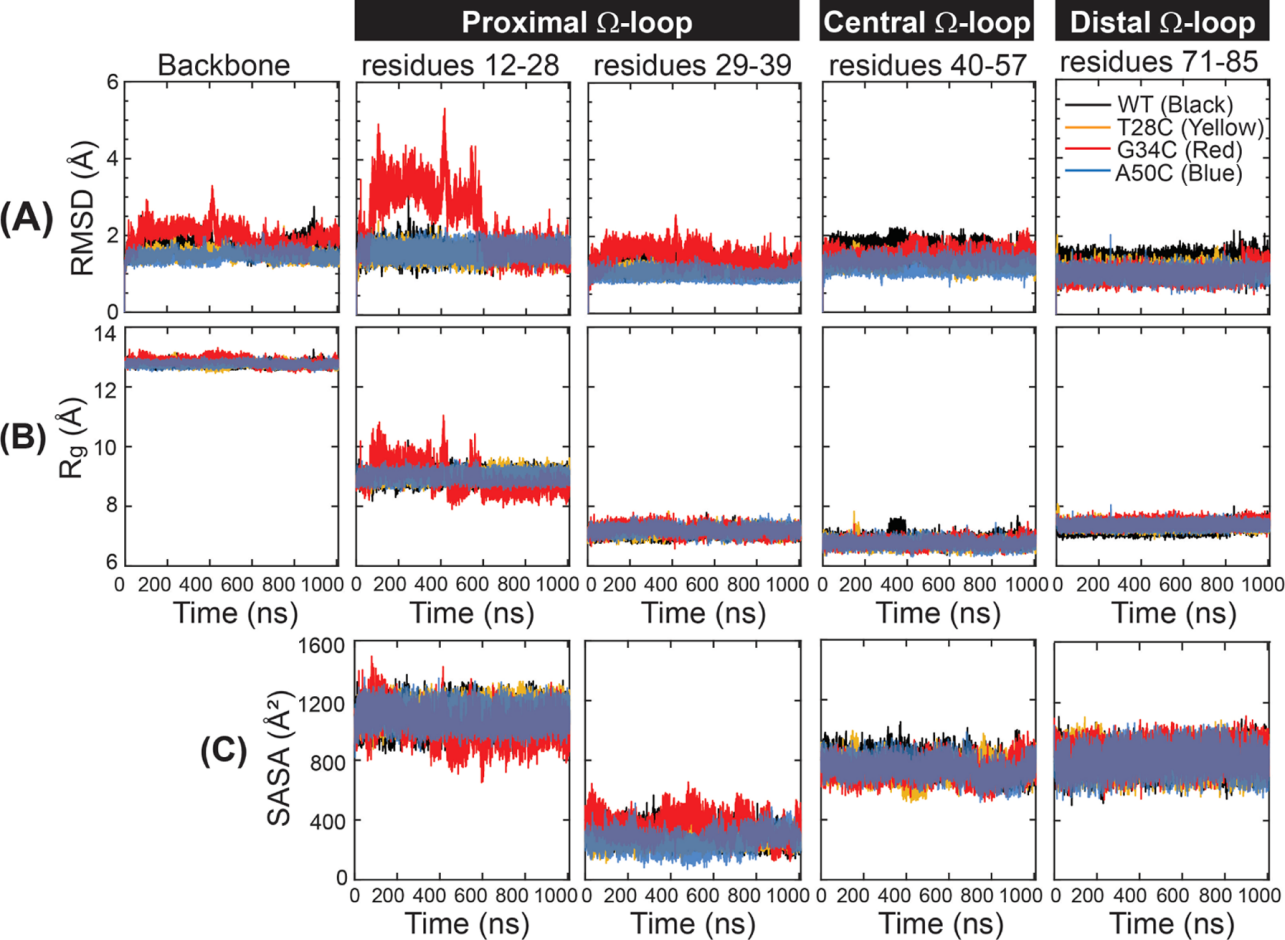
Time evolutions of 1000 ns MD simulations of WT (black), T28C (yellow), G34C (red), and A50C (blue) mutants, showing rmsd (A), Rg (B), and SASA (C) values in different regions of the protein structures.

The 1 μs MD simulations of the three mutants were performed in the oxidized (Fe(III)) state in aqueous solution. Note that the MD results of WT hCytc were derived from our previous report.^21^ In this study, structural stability and flexibility of each protein were determined by the rmsd, Rg, SASA, and rmsf calculations. The time evolutions of the MD parameters in different regions are shown in Figure 2, and their average values are listed in supplementary Table S2. In this study, in addition to the values derived from protein backbone atoms, the data were further calculated for individual Ω-loops to examine their specific structural changes during MD simulations. It should be noted that visual inspection of MD snapshots along the simulation time (data not shown) display the deformation of residues 12–28 of the proximal Ω-loop upon G34C mutation, which should be separately analyzed and discussed. Therefore, residues 12–39 of the proximal Ω-loop were divided into two segments, that is, residues 12–28 and 29–39 for further discussion.

The time evolutions of rmsd and Rg values are the fundamental features for monitoring protein stability and dynamics during the simulations,^26^ and they are commonly used to clarify the structural changes of the protein upon mutations. The rmsd plots (Figure 2A) of the protein backbone atoms, excluding the G34C system, are likely to reach equilibrium within 100 ns with the values averaged over 1000 ns of 1.75, 1.49, and 1.45 Å for the WT, T28C, and A50C hCytc, respectively (Table S2). The rather lower values for T28C and A50C mutants as compared to the WT suggest that the two mutants are slightly more stable. On the other hand, the G34C mutant exhibits the higher average rmsd value than that of the WT, particularly during ∼100–600 ns (rmsd value >2.5 Å, as discussed below). This is owing to the relatively high conformational flexibility at the proximal Ω-loop (residues 12–28), in which the rmsd at residues 12–28 is relatively greater than that of the same region for the remaining mutants and WT hCytc (Figure 2A). Obviously, the rmsd values of these residues for the G34C mutant (see the red line in the second column of Figure 2A) show the large fluctuation from ∼100 ns up to 600 ns, after which the value is uniformly fluctuated at ∼1.6 Å until the end of simulation. Therefore, the average rmsd during the entire timescale of simulation for residues 12–28 of the proximal Ω-loop becomes 2.44 Å with reasonably high standard deviations (SD) at 0.83 Å (see Table S2). It should be noted that for the proximal Ω-loop at residues 29–39, the rmsd of the G34C mutant is less fluctuated with 1.44 ± 0.25 Å but still higher than that of the same region of other systems. The possible explanation is that the G34C mutation could cause the local rearrangement of the surrounding residues and subsequently alter the hydrogen bond (H-bond) network in residues 12–28 of the proximal Ω-loop region. To monitor such an effect, the percentage of H-bond occupations is measured along the simulation time. The result shows that the H-bonds formed in the WT between Glu21—Asn31, Glu21—Gly24, and Thr19—Asn31 are disrupted in the G34C mutant (see Table S3 and Figure S1), thereby destabilizing the residues 12–28 of the proximal Ω-loop. The time evolution of the distance for the three relevant H-bonds was also calculated and plotted (Figure S2), showing the high fluctuation of these H-bond distances (∼3–8 Å), particularly for the distances between Glu21–Gly24 and Thr19–Asn31 during the first ∼600 ns of the G34C system.

In addition, the compactness of the protein structure can be measured by analysis of Rg determined from the root-mean-square average of the distance of all atoms from the center of mass of the protein. The high Rg value implies the protein structure with less tight packing and vice versa. From the plots of Rg in Figure 2B, the G34C mutant represents larger Rg variation among other systems during the simulation period. More specifically, the Rg values of individual Ω-loops in the G34C mutant obviously increase at residues 12–28 during the first 400 ns of the simulation, which is in line with the rmsd profiles. Consequently, the average Rg of G34C backbone atoms was found at 12.84 ± 0.11 Å, while those of the WT and other two mutants were almost identical at ∼12.76 ± 0.06 Å, indicating that these two mutations have no impact on the packing compactness of hCytc.

Additionally, the solvent accessibility is obtained from SASA analysis, which is defined as the surface area of the protein interacting with the surrounding solvent probe. Figure 2C highlights the SASA evolutions for all systems in four regions of hCytc. We found that the SASAs of the mutants and WT hCytc at residues 40–57 and 71–85 exhibit similar behavior with the average values of ∼726–771 and 800–835 Å^2^, respectively (see Table S2), which are consistent with the Rg data. However, the SASAs for residues 12–28 of the G34C mutant are not significantly increased during the first 400 ns, as seen in the Rg profile, which is most likely owing to the slight unfolding of this region (Figure S1), leading to the small changes in the water accessibility. Note that large fluctuation of SASA for the G34C mutant is observed within the first 400 ns, as shown in Figure S3. Whereas the distinctive feature of SASA observed at residues 29–39 of the G34C mutant (355 ± 69 Å^2^) is evidently greater than the other two mutants and WT (∼256–289 Å^2^), reflecting higher water accessibility to this region upon cysteine point mutation.

**Figure 3:**
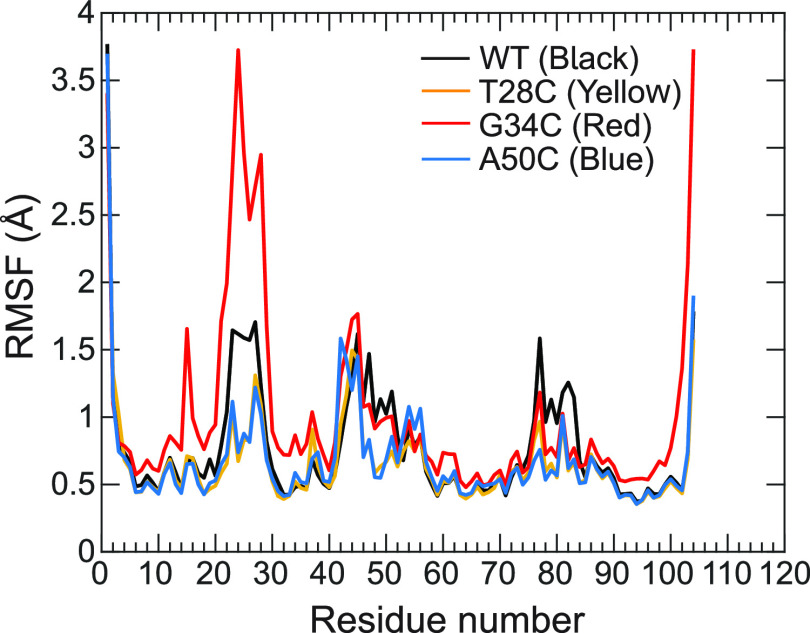
rmsf of *C*_α_ atoms as a function of amino acids for WT (black), T28C (yellow), G34C (red), and A50C (blue) mutants.

Finally, the analysis of rmsf, which measures the average deviation of the *C*_α_ atoms of each residue, is given in Figure 3. As expected, the three Ω-loop regions at residues 12–39, 40–57, and 71–85 display a higher rmsf than the α-helix regions for all mutants and WT hCytc. Besides, the distinctive rmsf pattern of the G34C mutant indicates increased conformational dynamics compared to the WT and other mutants of hCytc, particularly at the residues 21–29 (rmsf up to ∼3.7 Å). The result of rmsf is well consistent with the rmsd calculations, in which the less stable structure and larger conformational dynamics in the proximal Ω-loop region (residues 21–29) of the G34C mutant are observed as compared to WT hCytc. In comparison, the structural features of T28C and A50C mutants reveal the slightly lower structural deviation of the average rmsd at the backbone atoms than that of WT hCytc (Figure 2A). Furthermore, the local destabilization near the heme group with respect to initial structures can be predicted by the rmsd and rmsf at the 71–85 Ω-loop where the Met80 residue is located. The rmsf values around the Met80 region for G34C and other mutants are not significantly higher than those of the WT. In fact, they are slightly lower. The average rmsf at residues 71–85 were 0.9, 0.6, 0.7 and 0.6 for WT, T28C, G34C, and A50C, respectively, implying slightly less conformational dynamics than the WT, specifically for T28C and A50C, at this region. Likewise, the SDs of average rmsd at this region of the three mutants are in the similar range to that of WT hCytc, indicating that the heme group of all mutants is still stabilized when perturbed by the cysteine mutation. The similar rmsf result was also found in the MD study of WT horse heart cytc in comparison with Y67F and K72W mutants, in which the amino acid segment around the Met80 of the WT exhibited the slightly larger flexibility than that of the two mutants.^27^ Altogether, the rmsf and rmsd data at the 71–85 distal Ω-loop region agree with the model of the heme iron as the hexacoordinated form where the sixth ligand is covalently bound to Met80 along the simulation time. In general, it is acceptable that the peroxidase activity of cytc is induced by the dissociation of Met80 and, therefore, enables the access of substrates to the pentacoordinated heme iron. However, the classical model of the hexacoordinate form is thus utilized in our MD study to investigate the conformational dynamics of the precatalytic structures of hCytc.^9,28,29^ In this form, different structural dynamics are found in the G34C mutant, as suggested by rmsd, Rg, SASA, and rmsf calculations.

### 2.2 Structural Analyses Relating to Peroxidase Activity of hCytc

**Figure 4:**
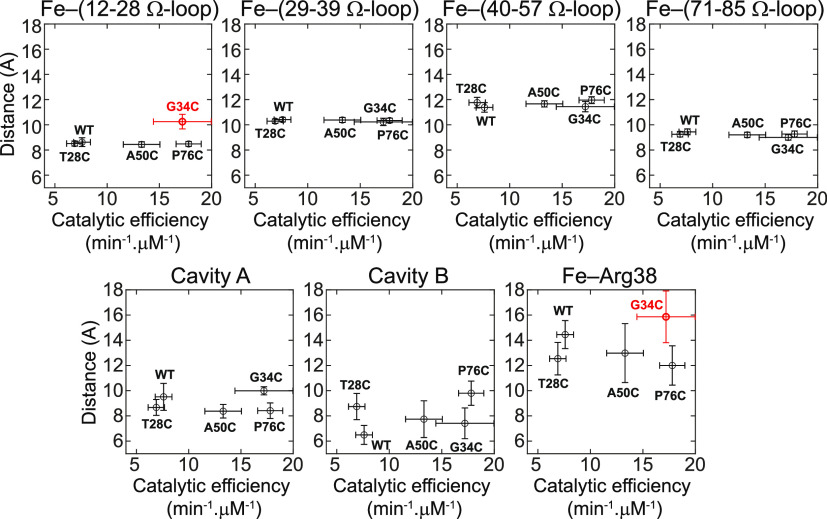
Plots showing correlation of catalytic efficiency to distances between heme iron and the center of mass of corresponding Ω-loops (upper panels (from left to right): Fe–(12–28 Ω-loop), Fe–(29–39 Ω-loop), Fe–(40–57 Ω-loop), and Fe–(71–85 Ω-loop)) and Cavity A (lower panel, left), Cavity B (lower panel, middle), and Fe–Arg38 (lower panel, right). Data of the WT and P76C were previously reported.^20,21^ The peroxidase activity was performed by ABTS assay when 2,2-azinobis(3-ethylbenthiazoline-6-sulfonic acid), ABTS, and H_2_O_2_ were used as substrates. Catalytic efficiency was calculated as /, where  and  are the Michalis–Menten parameters where the concentration of ABTS is varied in the ABTS assay.  is the maximum rate of reaction, and  is defined as the concentration of ABTS where the rate is half-maximum.

**Figure 5:**
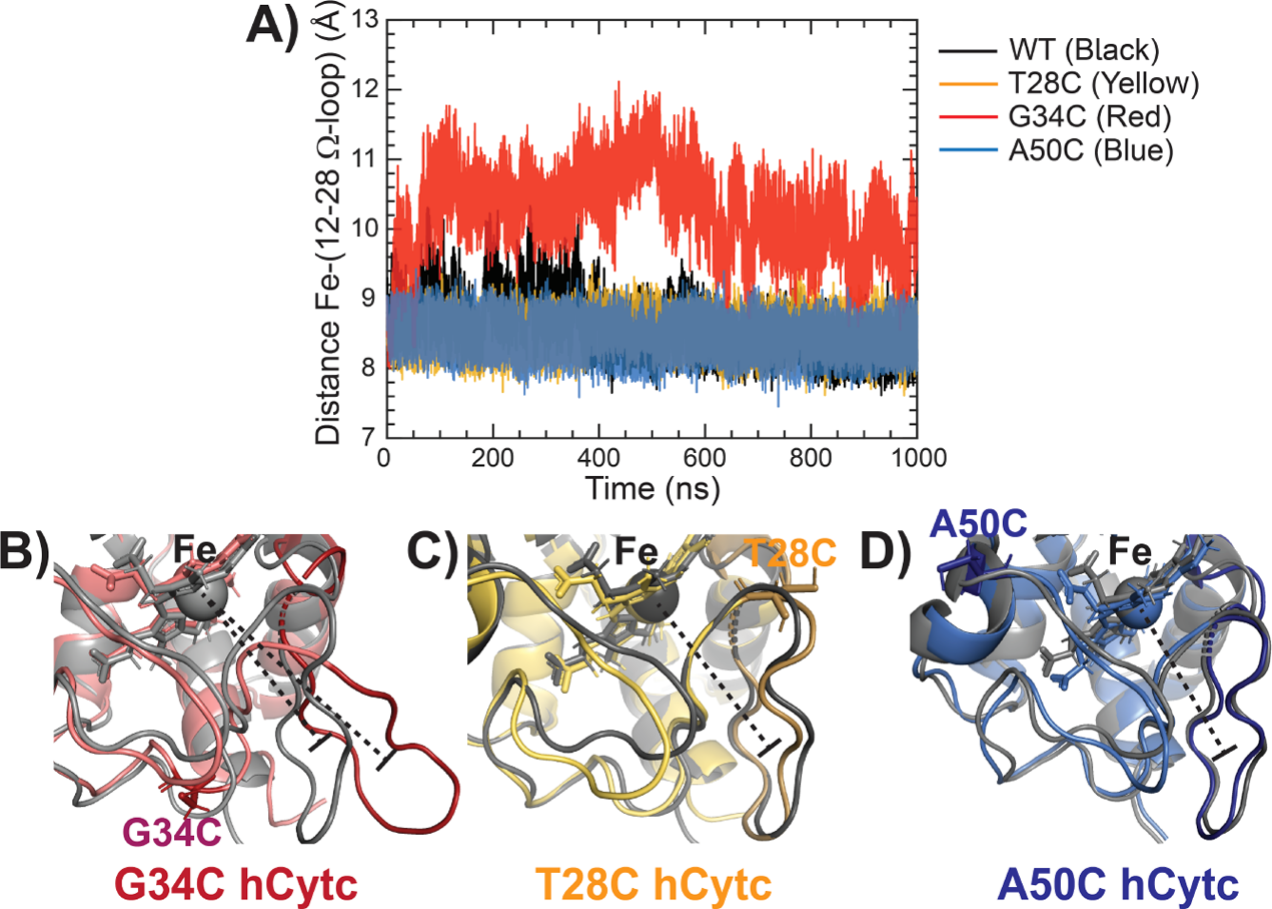
Time evolutions of the distances between Fe and the center of mass of the 12–28 Ω loop (A). Overlay of structures at 500 ns showing the distance between Fe and 40–57 Ω loop for the WT (gray) and G34C mutant (red) (B), WT (gray) and T28C mutant (yellow) (C), and WT (gray) and A50C mutant (blue) (D).

In this section, we aim to determine whether the structural dynamics at the Ω-loops of the hexacoordinated form may correspond to peroxidase activity observed in the hCytc mutants. The correlations with catalytic efficiency and the distances between the heme iron and the center of mass of each Ω-loop were observed (upper panel of Figure 4 and Table S1). For comparison, data of the P76C mutant from our previous work^21^ are included. According to the Pearson analysis in Table S4, the increased catalytic activity shows no association with the distances from heme iron to all individual Ω-loops, where the reported p-values are not significant. Besides, negative correlations were observed for the Fe–(29–39 Ω-loop) and Fe–(71–85 Ω-loop) distances. The preserved distances between the heme iron and each individual Ω-loops imply a stable conformation of the hexacoordinated form of hCytc upon perturbation by the mutations, which is in agreement with the rmsd, Rg and, SASA results (see Section 2.1). In contrast, the outlier in the plot of Fe–(12–28 Ω-loop) distance due to the G34C mutant, as shown in Figure 4, is worth to mention. The distance was more extended by ∼1.6 Å and larger distance fluctuation by ∼0.2 Å than that of WT and other mutants. The extended distance of Fe–(12–28 Ω-loop) in the G34C mutant as a function of simulation time is demonstrated in Figure 5A along with the representative structures of the mutants and WT hCytc taken from the 500th ns MD snapshot (Figure 5B–D). This precatalytic state of the G34C mutant is also different from that of the P76C mutant previously reported.^21^ Note that the enhancement of peroxidase activity of the P76C mutant was explained by the weakening of the H-bond between Tyr67 and Met80, whereas the H-bond distances between the two residues of T28C, G34C, and A50C mutants are relatively constant and comparable to the WT (∼3.3 Å, see Figure S4). Although there are no correlations between the catalytic efficiency and the abovementioned distances toward peroxidase activity of the hCytc mutants, the increase in flexibility at residues 12–28 of the proximal Ω-loop caused by G34C mutation is an intriguing issue that merits more research. More experimental investigation on mutations at this Ω-loop region is required to support our MD results in the future study.

**Figure 6:**
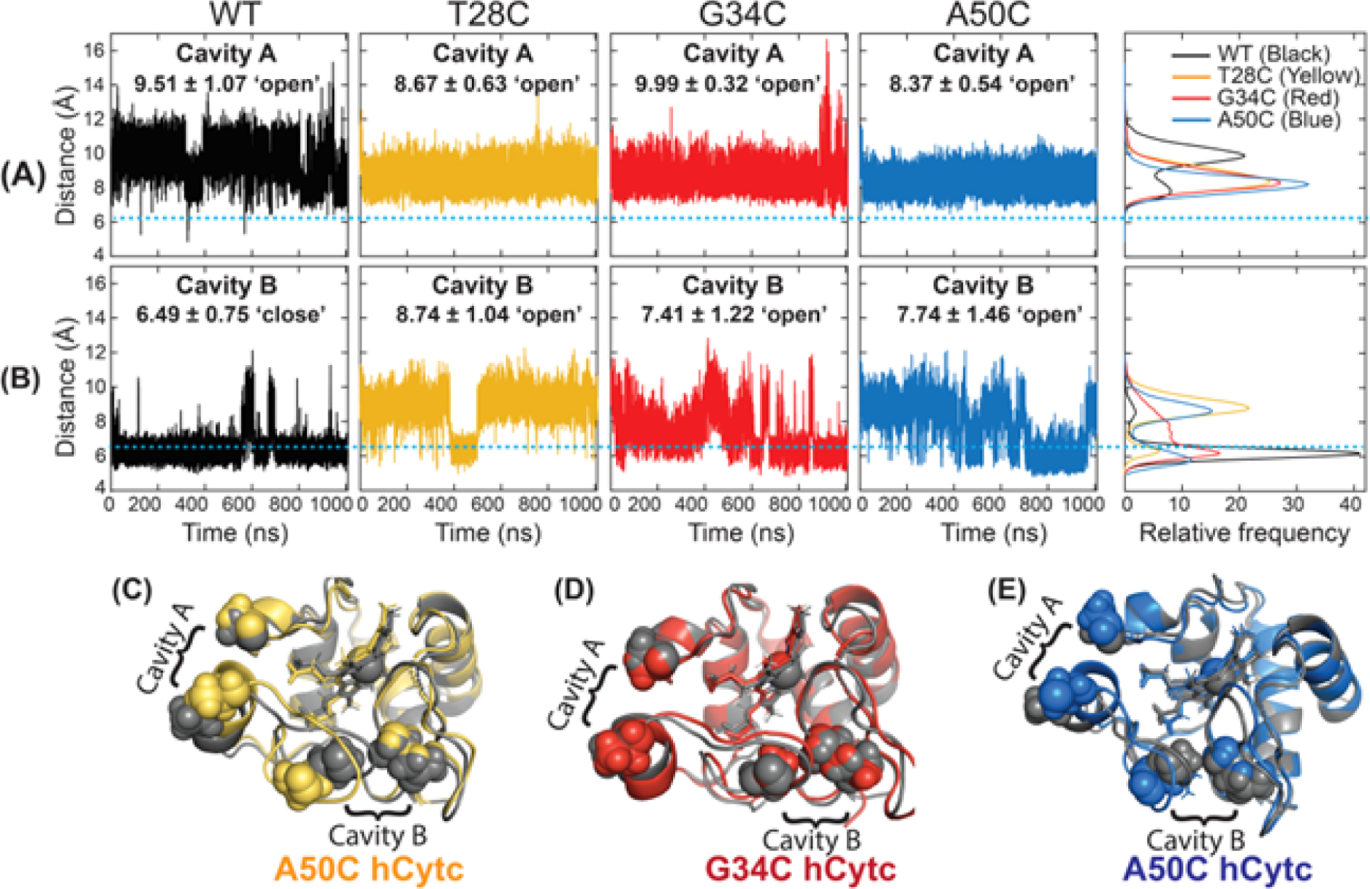
Distance variation of Cavity A (A) and Cavity B (B). The average distance and SD are reported on top of each plot. Distance distributions of *C*_α_–*C*_α_ distances obtained from the time evolutions are shown as the plot of frequency vs. distance (right column). The light blue lines represent cutoff distances of 6.2 and 6.5 Å for Cavity A and Cavity B, respectively. Overlay of MD snapshot at 900 ns showing the distances of Cavity A and Cavity B for the WT (gray) relative to the T28C mutant (yellow) (C), G34C mutant (red) (D), and A50C mutant (blue) (E).

We further performed distance analysis as previously implemented in our previous work on the P76C mutant. This calculation was proposed by Fellner et al.,^9^ in which the orientation of the Arg38 side chain with respect to the heme iron was investigated. Bortolotti et al.^30^ reported two distinctive fluctuating cavities in yeast iso-1-Cytc using a combination of redox properties and MD simulations. The formation of the two channels referred to Cavity A and Cavity B was defined by the residues belonging to the Ω-loops of the opposite sides, each facing one of the heme propionates. Cavity A was defined by residues 49–52 on one side and 76–79 on the other, whereas Cavity B was defined by residues 31–35 and 41–43. The movement of these cavities is considered to be relative displacements between the central and distal Ω-loops for Cavity A and between proximal and central Ω-loops for Cavity B. Therefore, the analysis of Cavity A and Cavity B can be measured by the time dependence of distances between *C*_α_ of A50–G77 and N31–A43, respectively.^30^ The reversible opening of the cavities was proposed to allow direct access of more water molecules to the heme crevice, also facilitating the access of H_2_O_2_ to the heme and initiating the peroxidase activity.^3,9,31^ Based on the abovementioned analyses, the time evolutions of Cavity A and B on the mutants and WT hCytc are provided in Figure 6 along with the distribution of the *C*_α_–*C*_α_ distance for each defined cavity on the right panel. Figure 6A shows that the variation on Cavity A as A50–G77 distance is higher in the WT, where all three mutants exhibit less deviations throughout the simulation time. The average distances of all systems are found in the “open” state (>6.2 Å), as defined by Bortolotti et al^30^ Simultaneously, the distance of Cavity B on the mutants in Figure 6B shows more variations than that of the WT as quantified by the SD values (Table S1). Cavity B of WT hCytc is dominantly located at ∼6.1 Å and spent most time in the “closed” state (<6.5 Å). The G34C mutant has the main distance centered at ∼6.2 Å and broader distribution at more than 6.5 Å. Both T28C and A50C mutants show bimodal distributions with the most probable distances about the same as ∼8.7 Å and less probability in the closing state with distances of 6.3 and 5.8 Å for T28C and A50C mutants, respectively. Figure 6C–E illustrates the superimposition between the WT and mutants, indicating the positions of Cavity A and Cavity B at 900th ns. Among all systems, the T28C mutant spends the most time in the opening state. The average distance of Cavity B is in the order T28C > A50C > G34C> WT hCytc. The results reveal that cysteine mutations at T28, A50, and G34 cause more impact on the degree and the movement of the opening of Cavity B when compared to the WT. However, the correlation of the peroxidase activity on the mutants is not straightforward. The non-active peroxidase T28C mutant shows more open distance, while less open distance of Cavity B is found in the increased peroxidase A50C and G34C mutants. By contrast, our previous data on the P76C mutant^21^ revealed that Cavity B stayed in the open state with the highest average distance (9.80 Å vs 6.49 Å of the WT), which correlated well with the most increased peroxidase activity.

**Figure 7:**
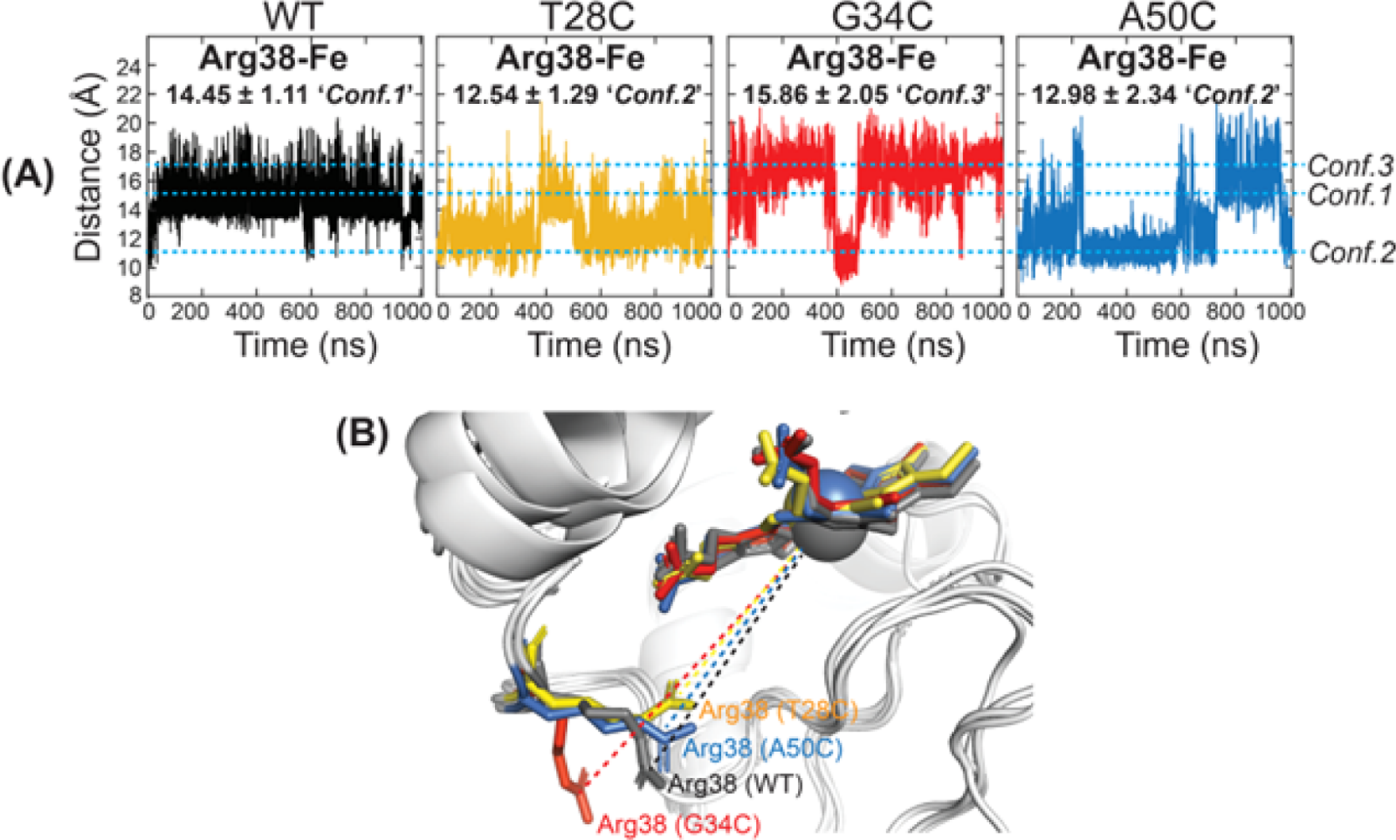
(A) Distance variation of Arg38(CZ)–Fe distance as a function of simulation time for WT, T28C, G34C, and A50C hCytc. The distances of Arg38(CZ)–Fe of 11, 15, and 17 Å are defined as Conf. 2, Conf. 1, and Conf. 3, respectively, as indicated by the dash blue lines. (B) Overlay of structures at 1000 ns showing orientation of Arg38 for the WT (gray), T28C (yellow), G34C (red), and A50C mutant (blue).

Next, our analysis on the role of the Arg38 side chain in peroxidase activity is depicted in Figure 7A as plots of the time evolution of the distance between Arg38 guanidino carbon (CZ) and heme iron. Previous studies on the crystal structure of yeast iso-1-cytc^7,9^ have characterized alternate positions of the Arg38 side chain that mediate the accessibility of water molecules to the heme prosthetic group. The Arg38 side chain can be classified into three conformers based on the analysis of Fellner et al^9^ The three possible conformers, denoted as Conf. 1, Conf. 2, and Conf. 3, are characterized by the distance between the CZ atom of Arg38 and heme iron as ∼15, ∼11, and ∼17 Å, respectively. It should be mentioned that the orientations of Arg38 of Conf. 1 and Conf. 3, facing toward the protein surface opposing the heme group, were found in the mutants with enhanced peroxidase activity.^6,9^ The average distance of Arg38(CZ)–Fe for the WT hCytc is found to be 14.45 Å, which is closer to Conf. 1 (∼15 Å). Note that the conformer found on the WT is in contrast with the crystal structure^7^ and recent MD result^9^ in which the Arg38 side chain was commonly oriented toward the heme iron corresponding to Conf. 2 (∼11 Å). The most probable distances of the mutants are found to be 11, 12, and 17 Å for A50C, T28C, and G34C mutants, respectively. In addition, the motion of Arg38 in the WT appeared to be more rigid than that in the mutants, as indicated from the smaller SD value (Table S1). In the case of the A50C mutant, the movement of the Arg38 side chain is mostly present as Conf. 2 at 11 Å (∼70%) and less probable at 15–17 Å (∼24%). Similarly, the T28C mutant has less probable distance of Arg38(CZ)–Fe at 14 Å (∼23%). Interestingly, the effect is evident in the G34C mutant where the side chain of Arg38 orients toward the protein surface as Conf. 3 at 17 Å (Figure 7B). This conformer was also observed in a peroxidase active mutant (G41S) of hCytc.^6^ Moreover, the high fluctuation of rmsd values at residues 12–28 of the proximal Ω-loop indicates the most flexible part in the G34C mutant (see Section 2.1). The data were further supported by analysis of the distance between Arg38 and (12–28 Ω-loop), as shown in Figure S5. The average distance in the G34C mutant was the most extended, and highest SD value was at 16.15 ± 1.91 Å vs. 14.21 ± 1.12 Å (WT), 12.86 ± 1.06 Å (T28C), and 13.71 ± 1.61 Å (A50C). The analysis of Arg38 orientation strongly supports the peroxidase enhancement in the G34C mutant in the precatalytic state. However, overall, statistical analysis indicates that the distances of Cavity A and Cavity B and Fe–Arg38 have no correlations to the catalytic efficiency (see the lower panel of Figure 4 and Table S4).

Taken together, the MD simulations suggest that the enhanced dynamic and conformational changes in the proximal Ω-loop may not contribute to the increased peroxidase activity for the G34C mutant. Likewise, our MD data with the A50C mutant do not support the correlation of increased peroxidase activity with the stabilized dynamics of all the Ω-loops. The inconsistency of the loop dynamics and the peroxidase activity was also found in the G41S mutant reported by MD simulations,^9^ where the active peroxidase structure of the G41S mutant exhibited lower overall flexibility than that of the WT. The MD data, therefore, suggested that the increased dynamics of the Ω-loops in the pre-catalytic state is not a pre-requisite for the increased peroxidase activity. Indeed, this mechanism of peroxidase activation would require the destabilization of the Fe-Met80 binding which may not be captured in the pre-catalytic state modeled in this study. Our MD data on the G34C mutant indicated that the pre-catalytic state where the Met80 is still bound to the heme iron was particularly destabilized in residues 12–28 of the proximal Ω-loop, and the Arg38 side chain is oriented toward the protein surface. The structural information obtained in the G34C mutant gives insights into the feasible effects of such a mutation on the proximal Ω-loop, in which the perturbation at the central and/or distal Ω-loop has been observed in the mutants on the distal Ω-loop.^10,18^

### 2.3 Conclusions

The different characteristics of structural information of hCytc obtained from the MD data allow us to describe its conformational switch between the multiple functions of the protein. Our 1 μs MD simulations characterize the folded structures of the cysteine mutants located in the proximal (T28C and G34C) and central (A50C) Ω-loops, modeled in hexacoordinated ferric iron referred to as a “precatalytic state”. In this form, distinctive structural dynamics are found in the G34C mutant, while mutations at T28C and A50C maintain normal folded states and are comparable to the WT as commonly observed in other mutants of Cytc.^9,32^ Analyses of each Ω-loop of the G34C mutant indicate the partial unfolding and less stable structure specifically in the proximal Ω-loop at residues 12–28 as suggested by rmsd and Rg calculations. Likewise, the rmsf values at this loop region show the most flexible movement when compared to the other two mutants and WT. The average distance between residues 12–28 of the proximal Ω-loop and heme iron is most extended, and H-bond disruptions in this loop region occurred in the G34C mutant. Recently, several reports focusing on natural mutants (G41S, Y48H, and A51V)^6,10,18,33^ of hCytc have elucidated the role of the central 40–58 Ω-loop on modulating the peroxidase activity and its interaction with the distal 71–85 Ω-loop through the H-bond network. Moreover, to be a peroxidase active form, the dynamics at the distal 71–85 Ω-loop is expected to be the most flexible since this loop region is close to the heme group and helps regulate the substrate accessibility to the heme iron.^34,35^ However, our MD results show that stability and flexibility at the distal 71–85 and central 40–58 Ω-loop of the three mutants do not significantly deviate from the WT. A similar result was also previously observed in the P76C mutant. It can be inferred that the sixth coordination of heme iron to Met80 is essential in stabilization of the normal folded state of Cytc. Hence, the impact of G34C mutation on the structural destabilization is striking. The results imply that the flexibility of the proximal Ω-loop, specifically residues 12–28, plays an important role in the precatalytic state upon cysteine mutation at Gly34. To the best of our knowledge, this is the first report of MD simulation results on the effect of mutations in the proximal Ω-loop.

To relate the structural information from our MD simulations to the experimental peroxidase activity of hCytc, the residues involved in enhanced accessibility to the heme are analyzed. We performed distance analysis on the opening/closing of two different channels identified as Cavity A and Cavity B. The behavior of Cavity B, defined by the N31–A43 *C*_α_ distance, observed in the three mutants is in the open state that shows a larger opening and more fluctuation than in the WT. Instead, the orientation of Arg38 conformers of the three mutants indicates more flexibility than the WT. The Arg38 side chain of the G34C mutant is oriented toward the protein surface and furthest away from the heme iron, feasibly facilitating the accessibility of the substrates, while the Arg38 of T28C and A50C mutants is pointing closer to the heme iron with respect to the WT. However, statistical analysis reveals that the distances of Cavity A and Cavity B and Fe–Arg38 show no correlations with the catalytic efficiency.

The MD results on the G34C mutant suggest the increased dynamics in the proximal Ω-loop, particularly at residues 12–28, but no relationship to the enhanced peroxidase activity is established. Similarly, the structural properties of the A50C mutant are similar to that of the WT, while its peroxidase activity is increased by ∼2.5 times of *k*_cat_, almost as high as that of the G34C mutant. This is most likely due to the ligation of Met80 to heme iron modeled in this study, indicating the influence of the rigidity of the distal 71–85 loop, which may not be influenced by the less stable proximal 12–39 and central 40–57 Ω-loops upon mutation. Overall, our MD results provide molecular insights into the degree of structural variations of hCytc upon the mutations. The precatalytic state of hCytc explained by MD simulations together with experimental data would offer complementary information to predict the mechanisms of multiple functions of hCytc and ultimately relate to the role of Ω-loops to the apoptotic function of hCytc.

## 3 Computational Methods

### 3.1 System Preparation

The computational methods were set up and performed using the same protocol in accordance with our previous study^21^ as follows. The crystal structure of WT hCytc was downloaded from the Research Collaboratory for Structural Bioinformatics Protein Data Bank (RCSB PDB)^36^ (PDB ID: 3ZCF(25)). To prepare the three mutants, T28C, G34C, and A50C, a single residue mutation of the WT hCytc structure (chain A) was carried out to change the native residue into the amino acid cysteine using the macromolecule tools implemented in Discovery Studio Visualizer (BIOVIA, San Diego, CA, USA). The heme prosthetic group in the oxidized state (Fe(III)) was assigned its partial atomic charges based on the theoretical study, as reported by Autenrieth et al^37^ To derive the bonded and nonbonded parameters for a heme group in a bonded model, a python-based metal center parameter builder, MCPB.py module^38^ of AMBER16, was employed. The general AMBER force field (GAFF)^39^ and the AMBER ff14SB^40^ force fields were adopted to treat the heme group and hCytc, respectively. The ionizable residues (Asp, Glu, Lys, Arg, and His) were considered their protonation states based on the prediction from the H++ web server^41^ at pH 7.0. Next, each system was electrostatically neutralized by the counterions Cl^–^ and solvated using the TIP3P water model^42^ with a minimum distance between the solvation box edge and protein surface of 10 Å. The added hydrogen atoms and solvent molecules were minimized using 1000 iterations of steepest descent followed by 2000 iterations of conjugated gradient methods. Finally, the entire system was energetically minimized utilizing the same minimization procedure.

### 3.2 Molecular Dynamics Simulation

Each simulated system was run under periodic boundary conditions with the isobaric-isothermal (*NPT*) ensemble (300 K and 1 bar) using pmemd.cuda in AMBER16. A 10 Å distance cutoff was applied for the short-range nonbonded interactions, while the long-range electrostatic interactions were treated using the Particle Mesh Ewald (PME) algorithm.^43^ To maintain all covalent bonds involving hydrogen atoms, the SHAKE algorithm^44^ was utilized, allowing an integration time step of 2 fs. The target temperature and pressure were retained using the Langevin thermostat with a collision frequency of 2 ps^–1^^45^ and the Berendsen barostat^46^ with a pressure relaxation time of 1 ps, respectively. Initially, each model was slowly heated up from 10 to 300 K for 200 ps with the application of a harmonic restraint of 30.0 kcal·mol^–1^·Å^–2^ to the *C*_α_ atoms of the protein. In the following equilibrium step, the systems were subjected to restrained MD simulations at 300 K with decreasing harmonic restraint of 30, 20, 10, and 5 kcal·mol^–1^·Å^–2^ for 1300 ps, continued by unrestrained MD at the target temperature for another 200 ps. In the production phase, MD simulations were simulated under the *NPT* scheme until 1000 ns was reached. The CPPTRAJ module^47^ was employed to compute structural analyses, including rmsd, Rg, SASA, rmsf, and distance analysis on important residues. For H-bond occupation analysis, a H-bond was considered to be present if the following geometric criteria were achieved: (i) a hydrogen donor (HD)–acceptor (HA) distance of ≤3.5 Å and (*ii*) a HD–H···HA angle of ≥120°.

## Supporting Information Available

Comparison of each defined distance over 1000 ns MD simulations for WT, T28C, A50C, and G34C hCytc and the corresponding peroxidase activity and rotational correlation time of each system; average values and corresponding standard deviations of each structural parameter for various regions of WT, T28C, A50C, and G34C hCytc calculated from 1000 ns MD simulation trajectories; comparison of hydrogen bond occupations in the 12–39 proximal Ω-loop between the WT and G34C mutant during MD simulations; comparison of the structure in the 12–39 Ω-loop of the WT and G34C mutant at the 1000 ns snapshot; time evolution of the distances relevant to the H-bonding network at the residues 12–28 for the WT and G34C mutant; time evolution of SASA values calculated at the residue 12–28 region; statistical analysis of the relationship between the indicated distances and the peroxidase catalytic efficiency of hCytc systems; time evolution of the distances between Met80 and Tyr67 for all systems of hCytc; and time evolutions of the distances between the CZ atom of Arg38 and the 12–28 Ω-loop (PDF)
