# Supplementary material for: Structural Dynamics of the Precatalytic State of Human Cytochrome c upon T28C, G34C, and A50C Mutations: A Molecular Dynamics Simulation Perspective
Source: ACS Omega. 2023 Apr 17;8(17):15229–38. doi: 10.1021/acsomega.3c00220 (PMC10157674; doi:10.1021/acsomega.3c00220)
Supplement: Supplementary file 1 — ao3c00220_si_001.pdf [file ao3c00220_si_001.pdf]

Supporting Information

Structural dynamics of the precatalytic state of human cytochrome c upon T28C, G34C and A50C mutations: A molecular dynamics simulation perspective

Bodee Nutho[a], Sasiprapa Samsri[b], Soraya Pornsuwan[b*]

[a] Department of Pharmacology, Faculty of Science, Mahidol University, Bangkok, 10400, Thailand.

[b] Department of Chemistry and Center of Excellence for Innovation in Chemistry, Faculty of Science, Mahidol University, Bangkok, 10400, Thailand.

Table S1. Comparison of each defined distance over 1000-ns MD simulations for WT, T28C, A50C and G34C hCytc and the corresponding peroxidase activity and rotational correlation time of each system.

	**WT (Å)**	**T28C (Å)**	**G34C (Å)**	**A50C (Å)**	**P76C (Å)**
**Distances between the center of mass of indicated Ω loop and heme iron, Fe**					
Ω loop 12-28 – Fe	8.62 ± 0.35[a]	8.51 ± 0.20	10.25 ± 0.58	8.44 ± 0.21	8.48 ± 0.19[a]
Ω loop 29-39 – Fe	10.41 ± 0.18[a]	10.29 ± 0.18	10.23 ± 0.29	10.38 ± 0.20	10.34 ± 0.18[a]
Ω loop 40-57 – Fe	11.38 ± 0.38[a]	11.76 ± 0.42	11.44 ± 0.41	11.66 ± 0.25	11.96 ± 0.27[a]
Ω loop 71-85 – Fe	9.44 ± 0.23[a]	9.25 ± 0.22	9.00 ± 0.22	9.20 ± 0.21	9.27 ± 0.24[a]
**Distances between two indicated residues**					
**Cavity A**					
Ala50(C_α) – Gly77(C_α)	9.51 ± 1.07[a]	8.67 ± 0.63	9.99 ± 0.32	8.37 ± 0.54	8.41 ± 0.62[a]
**Cavity B**					
Asn31(C_α) – Ala43(C_α)	6.49 ± 0.75[a]	8.74 ± 1.04	7.74 ± 1.46	7.41 ± 1.22	9.80 ± 0.96[a]
Arg38(CZ) – Fe	14.45 ± 1.11[a]	12.54 ± 1.29	15.86 ± 2.05	12.98 ± 2.34	12.00 ± 1.56[a]
**Peroxidase activity***					
k_{cat}^{ABTS} (min^{-1})**	17.7 ± 0.3[b]	17.7 ± 0.0[b]	46.4 ± 0.6[b]	44.3 ± 0.4[b]	229.9 ± 4.6[a]
K_M^{ABTS} (μM)**	2.3 ± 0.2[b]	2.6 ± 0.3[b]	2.7 ± 0.4[b]	3.3 ± 0.4[b]	12.9 ± 0.6[a]
Catalytic efficiency ($min^{-1}.\mu M^{-1}$)***	7.6 ± 0.80[b]	6.9 ± 0.78[b]	17.2 ± 2.77[b]	13.3 ± 1.75[b]	17.8 ± 1.2[a]
Rotational correlation time, τ_R (ns)****	-	5.20 ± 0.03[b]	3.17 ± 0.02[b]	3.44 ± 0.02[b]	2.06 ± 0.02[a]

[a]Data were previously reported in reference 1[1]

[b]Data were previously reported in reference 2[2]

* Peroxidase activity was performed by ABTS assay when 2,2-Azinobis(3-ethylbenthiazoline-6-sulfonic acid), ABTS, and H_2O_2 were used as substrates.

** k_{cat}^{ABTS} and K_M^{ABTS} are the Michalis-Menten parameters where the concentration of ABTS is varied in the ABTS assay. The k_{cat}^{ABTS} is the maximum rate of reaction and K_M^{ABTS} is defined as the concentration of ABTS where the rate is half maximum.

*** Catalytic efficiency was calculated as $k_{cat}^{ABTS}/K_M^{ABTS}$.

**** Rotational correlation time, τ_R, was determined by the simulation of ESR spectra using EasySpin package[3]. The higher τ_R value, the slower rotational rate at the spin-labeled site, and *vice versa*.

Table S2. Average values and corresponding standard deviations of each structural parameter for various regions of WT, T28C, A50C, and G34C hCytc calculated from 1000-ns MD simulation trajectories.

**Properties**	**WT hCytc**	**T28C hCytc**	**G34C hCytc**	**A50C hCytc**
**Average RMSD (Å)**				
Backbone	1.75 ± 0.15[a]	1.49 ± 0.11	1.96 ± 0.26	1.45 ± 0.10
12-28 Ω-loop	1.59 ± 0.16[a]	1.58 ± 0.14	2.44 ± 0.83	1.64 ± 0.14
29-39 Ω-loop	1.25 ± 0.11[a]	1.11 ± 0.09	1.44 ± 0.25	0.99 ± 0.09
40-57 Ω-loop	1.63 ± 0.19[a]	1.25 ± 0.13	1.49 ± 0.14	1.20 ± 0.11
71-85 Ω-loop	1.31 ± 0.15[a]	1.13 ± 0.11	1.03 ± 0.16	1.04 ± 0.10
**Average Rg (Å)**				
Backbone	12.77 ± 0.07[a]	12.76 ± 0.07	12.84 ± 0.11	12.76 ± 0.06
12-28 Ω-loop	9.02 ± 0.15[a]	9.01 ± 0.13	8.94 ± 0.44	9.00 ± 0.12
29-39 Ω-loop	7.09 ± 0.10[a]	7.18 ± 0.11	7.20 ± 0.14	7.19 ± 0.12
40-57 Ω-loop	6.86 ± 0.15[a]	6.75 ± 0.12	6.77 ± 0.11	6.74 ± 0.11
71-85 Ω-loop	7.22 ± 0.12[a]	7.35 ± 0.10	7.41 ± 0.10	7.36 ± 0.08
**Average SASA (Å^2)**				
12-28 Ω-loop	1098 ± 67[a]	1111 ± 57	1034 ± 97	1102 ± 54
29-39 Ω-loop	289 ± 44[a]	267 ± 37	355 ± 69	256 ± 55
40-57 Ω-loop	771 ± 57[a]	731 ± 59	726 ± 54	739 ± 56
71-85 Ω-loop	803 ± 65[a]	812 ± 70	835 ± 71	799 ± 74
**Average RMSF (Å)**	0.78 ± 0.46[a]	0.66 ± 0.36	1.01 ± 0.70	0.68 ± 0.40

[a]Data previously reported in reference 1[1]

Table S3. Comparison of hydrogen bond occupations in the 12-39 proximal Ω-loop between WT and G34C mutant during MD simulations.

Acceptor	DonorH	% Occupancy[*]	
		WT	G34C mutant
Pro30@O	His18@HD1	99.99	99.80
Thr19@O	Leu32@H	99.57	96.29
Cys14@O	Cys17@H	95.70	65.47
Glu21@O	Asn31@HD21	90.46	–
Leu35@O	Arg38@H	89.99	71.85
Asn31@OD1	His33@H	80.13	86.42
Leu32@O	Leu35@H	80.01	61.05
Glu21@O	Gly24@H	66.63	–
Thr19@OG1	Asn31@HD22	56.06	–
Gln16@OE1	Gln16@H	–	61.56

[*] The % H-bond occupations with more than 50% are reported.
'–' represents that no hydrogen bond was observed.

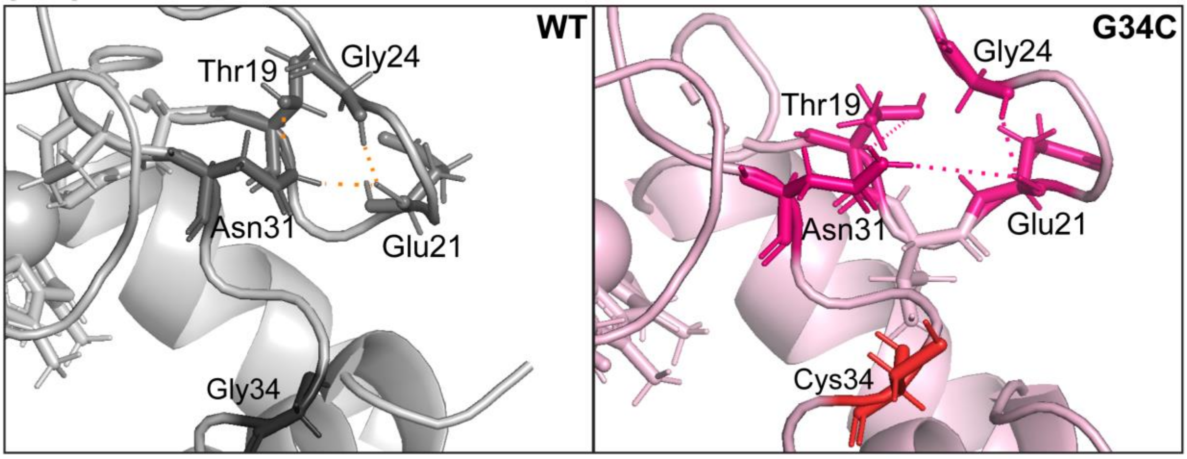

Figure S1. Comparison of the structure at the 12-39 Ω-loop of WT (left) and G34C mutant (right) at the 1000 ns snapshot. The H-bonds between Glu21 – Asn31, Glu21 – Gly24 and Thr19 – Asn31 observed in WT are shown in orange dotted lines, whereas these H-bonds were not observed in G34C. The pink dotted lines indicate the distances between residues forming H-bonds in WT. Residue 34 is shown as Gly34 for WT and Cys34 for G34C mutant.

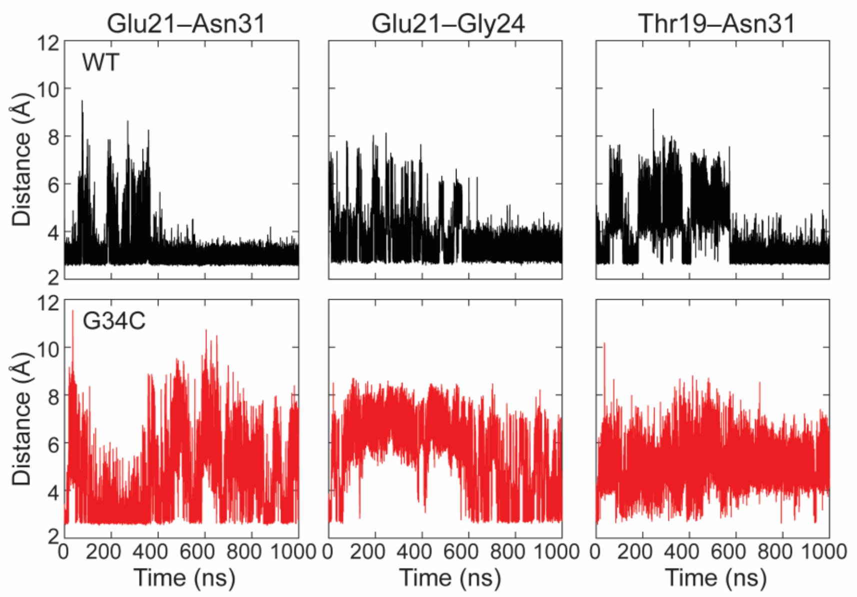

Figure S2. Time evolution of the distances relevant to the H-bonding network at the residues 12-28 for WT (upper panel) and G34C mutant (lower panel).

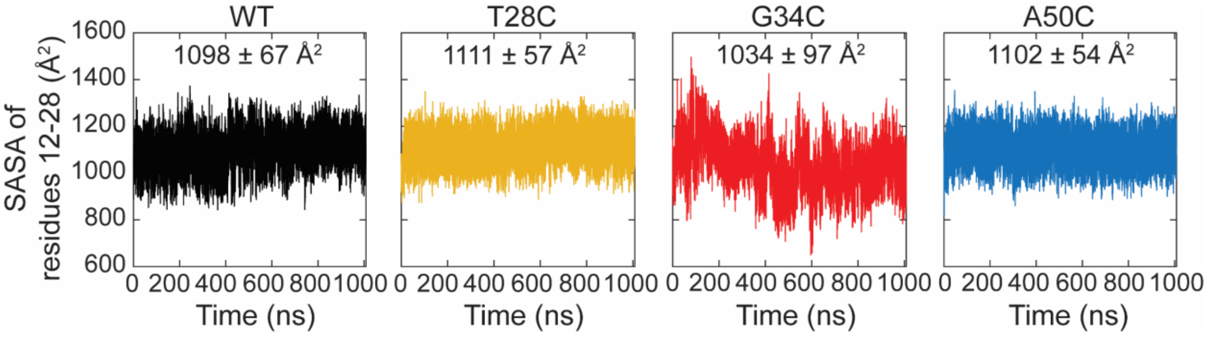

Figure S3. Time evolution of SASA values calculated at residues 12-28 region for WT (black line), T28C (yellow line), G34C (red line), and A50C (blue line) mutants. The average and SD of SASA values of each hCytc system are shown on top of the plots.

Table S4. Statistical analysis of the relationship between the indicated distances and peroxidase catalytic efficiency of hCytc systems.

**Distance**	**r***	**p-value**
Fe–(12-28 Ω-loop)	0.45	0.44
Fe–(29-39 Ω-loop)	-0.37	0.54
Fe–(40-57 Ω-loop)	0.28	0.65
Fe–(71-85 Ω-loop)	-0.62	0.25
A50–G77 (Cavity A)	0.02	0.97
N31–A43 (Cavity B)	0.37	0.53
Fe–Arg38	0.10	0.86

* r = Pearson correlation. Pearson analyses were performed with number of data = 5, and degree of freedom = 3. The threshold p-value = 0.05.

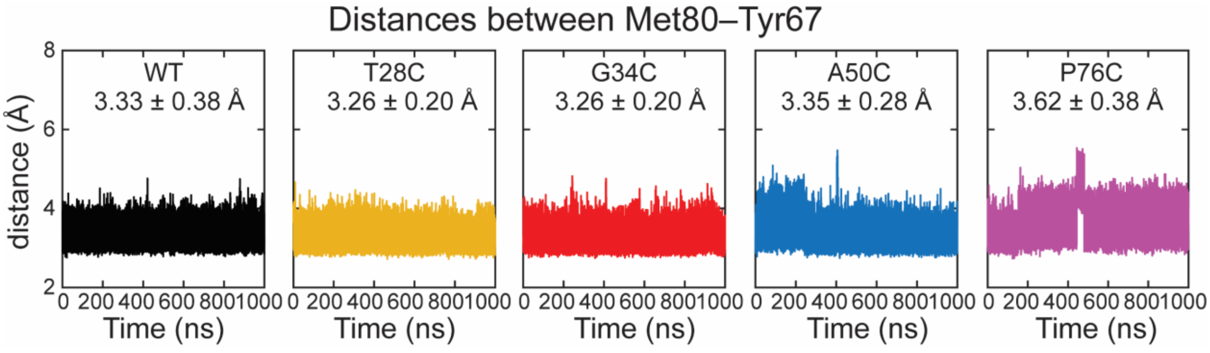

Figure S4. Time evolution of the distances between Met80 and Tyr67 for all systems of hCytc. The average distance and corresponding SD are reported on top of each plot. Note that the data of WT and P76C mutant were previously reported in reference 2.

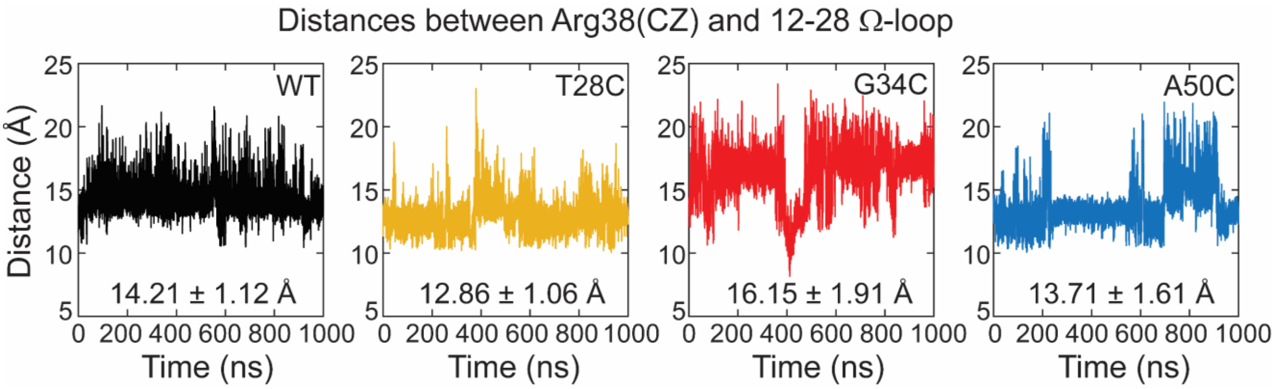

Figure S5. Time evolutions of the distances between the CZ atom of Arg38 and 12-28 Ω-loop of WT, T28C, G34C and A50C hCytc systems. The average distances and SD values of each hCytc system are shown at the bottom of the plots.

References:

1. Samsri, S.; Prasertsuk, P.; Nutho, B.; Pornsuwan, S., Molecular insights on the conformational dynamics of a P76C mutant of human cytochrome c and the enhancement on its peroxidase activity. *Arch. Biochem. Biophys.* **2022,** *716,* 109112.

2. Samsri, S.; Pornsuwan, S., Influence of cysteine-directed mutations at the Omega-loops on peroxidase activity of human cytochrome c. *Arch. Biochem. Biophys.* **2021,** *709*, 108980.

3. Stoll, S.; Schweiger, A., EasySpin: Simulating cw ESR spectra. *Biol. Magn. Reson.* **2007,** *27*, 299-321.
